# Dysfunctional phenotypes of CD4+ and CD8+ T cells are comparable in patients initiating ART during early or chronic HIV-1 infection

**DOI:** 10.1097/MD.0000000000003738

**Published:** 2016-06-10

**Authors:** Sylvie Amu, Rebecka Lantto Graham, Yonas Bekele, Aikaterini Nasi, Carina Bengtsson, Bence Rethi, Sam Sorial, Genny Meini, Maurizio Zazzi, Bo Hejdeman, Francesca Chiodi

**Affiliations:** a Department of Microbiology, Tumor and Cell Biology; b Department of Medicine at Solna; c Department of Clinical Science and Education, Södersjukhuset, Karolinska Institutet and Unit of Infectious Diseases/Venhälsan, Södersjukhuset, Stockholm, Sweden; d Department of Microbiology and Virology, Policlinico S. Maria alle Scotte, Siena, Italy.

**Keywords:** activation, differentiation, early ART, HIV-1, immunoactivation, inflammation markers, T-cell phenotype, virus reservoirs

## Abstract

Early initiation of antiretroviral therapy (ART) is becoming a common clinical practice according to current guidelines recommending treatment to all HIV-1-infected patients. However, it is not known whether ART initiated during the early phase of infection prevents the establishment of abnormal phenotypic features previously reported in CD4+ and CD8+T cells during chronic HIV-1 infection. In this cross-sectional study, blood specimens were obtained from 17 HIV-1-infected patients who began ART treatment shortly after infection (early ART [EA]), 17 age-matched HIV-1-infected patients who started ART during chronic phase of infection (late ART [LA]), and 25 age-matched non-HIV-1-infected controls. At collection of specimens, patients in EA and LA groups had received ART for comparable periods of time. Total HIV-1 DNA was measured in white blood cells by quantitative PCR. The concentration of 9 inflammatory parameters and 1 marker of fibrosis, including sCD14 and β-2 microglobulin, was measured in plasma. Furthermore, expression of markers of abnormal immune activation (human leukocyte antigen - antigen D related [HLA-DR] and CD38), exhaustion (programmed death 1, CD28, CD57) and terminal differentiation (CD127) was measured on CD4+ and CD8+T cells. T-cell proliferation was measured through Ki67 expression. The copies of total HIV-1 DNA in blood were significantly lower (*P* = 0.009) in EA compared with that in LA group. Only the expression of HLA-DR on naïve CD4+ T cells distinguished EA from LA, whereas expression of 3 surface markers distinguished T-cell populations of HIV-1-infected patients from controls. These included HLA-DR distinguishing CD4+ T cells from EA compared with controls, and also CD38 and CD127 on CD4+ and CD8+ T cells, respectively, distinguishing both groups of patients from controls. The sCD14 levels were significantly higher in EA patients, and β-2 microglobulin levels were higher in LA group compared with that in controls. Our results demonstrate an equivalent abnormal expression of activation (HLA-DR and CD38 on CD4+ T cells) and terminal differentiation (CD127 on CD8+ T cells) markers in T cells from both EA and LA patients. The size of total HIV-1 DNA copies in blood of EA was lower compared with LA patients. These findings suggest that some abnormalities taking place in the T-cell compartment during primary HIV-1 infection may not be corrected by early ART.

## Abstract 2

Supplemental Digital Content is available in the text

## 1 Introduction

Introduction of antiretroviral therapy (ART) has led to a substantial improvement in quality of life and life-length expectancy for HIV-1-infected patients. It has recently been shown that initiation of ART in HIV-1-infected patients during the early asymptomatic stage of infection, defined by a CD4+ T-cell count of more than 500 cells/μL, compared with patients who received ART when their CD4+ T-cell count decreased to 350 cells/μL, provided a net clinical benefit (Insight START Study group 2015).^[1]^ This has been quantified through a cumulative clinical primary end point, gathering serious AIDS-related events together with non-AIDS-related events. Short-course ART during primary HIV-1 infection (PHI) (Short Pulse Anti-Retroviral Therapy at Seroconversion Trial) also delayed the outcome of a primary end point, measured as CD4+ T-cell count less than 350 cells/μL or subsequent long-term ART initiation.^[2]^

In addition to the important clinical benefit resulting from early initiation of ART, the effect on reducing HIV-1 reservoirs is being investigated. It has been suggested that the initiation of ART as early as a few weeks postinfection may lead to the establishment of smaller-size HIV-1 reservoirs, as compared with the reservoirs established in patients initiating treatment during the chronic phase of infection.^[3]^ Initiation of ART during primary PHI, followed by interruption of therapy, was shown to lead, in a minority of individuals, to transient aviremic remission^[4,5]^ and sustained posttreatment control.^[3]^

A large variety of phenotypic dysfunctions occurring in both CD4+ and CD8+ T cells has been associated with HIV-1 infection, affecting both HIV-1-specific and non-HIV-1-specific T cells. These pathological findings are ameliorated but not fully corrected by ART. The phenotypic dysfunctions include distinct or combined features of abnormal immune activation, senescence, and inhibition of immune responses. The down-regulation of CD28 on T cells characterizes antigen-experienced cells at late stage of differentiation, where CD28− T cells are expanded in aged individuals,^[6]^ patients with diverse autoimmune disorders,^[7,8]^ and during HIV-1 infection.^[9,10]^ Expression of CD57 has also been shown to be a valuable marker for T-cell senescence in HIV-1-infected individuals.^[10,11]^ A decrease in CD127 (IL-7 receptor alpha) expression has previously been reported to occur on both CD4+ and CD8+ T cells from HIV-1-infected patients,^[12,13]^ where low CD127 expression may limit the effect of IL-7 on T-cell survival and homeostatic proliferation. The co-inhibitory receptor programmed death 1 (PD-1) has been shown to be highly expressed on HIV-1-specific and simian immunodeficiency virus (SIV)-specific CD8+ T cells.^[14,15]^ Cytokine production and proliferative capacity of CD8+ T cells during SIV infection is enhanced upon in vitro PD-1 blockade.^[16]^ Immunological abnormalities associated with HIV-1 infection also comprise chronic immune activation which has been measured by increased expression of human leukocyte antigen - antigen D related (HLA-DR) and CD38 markers on T cells.^[17,18]^ The chronic immune activation that is considered to be a driving force of HIV-1 pathogenesis is reduced, but not completely normalized, during ART.^[19,20]^

HIV-1 infection is associated with chronic systemic inflammation possibly related to microbial translocation through the damaged epithelial barrier in the gut.^[21]^ This inflammatory response manifests by an excess risk of cardiovascular, bone, liver, kidney, and bone diseases.^[22]^ Furthermore, inflammation markers, strongly predictive of the risk of morbidity and mortality, are elevated in circulation during HIV-1 infection.

The cross-sectional study described herein includes 2 groups of HIV-1-infected patients who had previously begun ART either at an early time point after infection or during a more chronic phase. The aim of this study was to investigate whether early ART (EA) initiation prevents the establishment of abnormal phenotypic features reported to take place on CD4+ and CD8+ T cells and whether differences in T-cell phenotype could be detected in the 2 groups according to time of treatment initiation. The copies of total HIV-1 DNA, as a measure of the virus reservoir, were determined in peripheral blood mononuclear cells (PBMCs) and compared with frequencies of T-cell subpopulations. As administration of ART early after infection may control fenestration of the epithelial barrier, and reduce microbial translocation, and also immune activation, parameters of inflammation were assessed.

## 2 Methods

### 2.1 Patient groups

Blood specimens were obtained from 17 (16 males [M] and 1 female [F]) HIV-1-infected patients who started ART treatment early after infection (EA) (age mean and standard deviation [SD] 43.8 ± 14.9 years) and 17 (16 M and 1 F) HIV-1-infected patients who began ART treatment during the chronic phase of infection (late ART [LA]) (age 42 ± 11.2 years). At the time of specimen collection, patients in the EA and LA groups had received ART for a comparable period of time; individuals in the EA group were treated for a median of 25 months (range 7–59) and individuals in the LA group were treated for a median of 29 months (range 12–60). Twenty-five healthy controls (all M) (defined as C) [age 40.4 ± 14.0] were selected as controls.

The first weeks after HIV-1 infection can be divided into distinct clinical stages characterized by a stepwise rise in assays positive for HIV-1-specific antibodies and HIV-1 antigens, the so called Fiebig stages I to V.^[23]^ In our cohort of early treated patients, 11 were classified as Fiebig stage II, 3 as stage IV, and another 3 patients as stage V. In the latter group of 3 individuals, all initiated ART within 7 weeks from the debut of a symptomatic acute HIV-1 infection and/or an earlier negative HIV test.

First ART regime included 2 nucleoside reverse transcriptase inhibitors (NRTIs) as backbone in all patients. Protease inhibitors (PIs) were added in 8 patients in the EA group and 6 patients in the LA group. Integrase inhibitors (INIs) were added in 6 patients in the EA group and in 7 patients in the LA group, and for the rest of the patients, non-nucleoside reverse transcriptase inhibitors (NNRTIs) were used. In 4 patients in the EA group, a fourth drug with CCR5 inhibitors was used until HIV-1 RNA reached down below detection (<20 copies/mL). At sampling, NRTIs continued to be the backbone in all except 1 patient in the LA group. Two patients in the EA group and 6 patients in the LA group continued with PIs. Twelve patients in the EA group and 9 in the LA group had a regime including NNRTIs. INIs were used in 2 patients in the EA group and 7 patients in the LA group.

Only 1 patient in the LA group had a coinfection with hepatitis B, but this was treated and under good control at sampling. One patient in the EA group and 2 in the LA group were treated with angiotensin-converting-enzyme inhibitors for hypertension, and a similar number of patients were treated with statins in each group. Only 1 patient in the LA group was treated with a low dose of acetylsalicylic acid (ASA). No steroids or other immune-modulating drugs were used in the patients included in the study.

Plasma HIV-1 RNA load was measured by Cobas Amplicor (Roche Molecular Systems, Branchburg, Inc., NJ). At the time of ART initiation the EA group had, as expected, a higher virus titer, with a HIV-1 RNA value (copies/mL) of 5.97 log as compared with 4.57 log in the LA group (*P* < 0.01). At 12 months from the initiation of ART and at the time of sampling of blood specimens for the present study, the virus was undetectable (<20 copies/mL) in the blood of all HIV-1-infected individuals.

**Figure 1:**
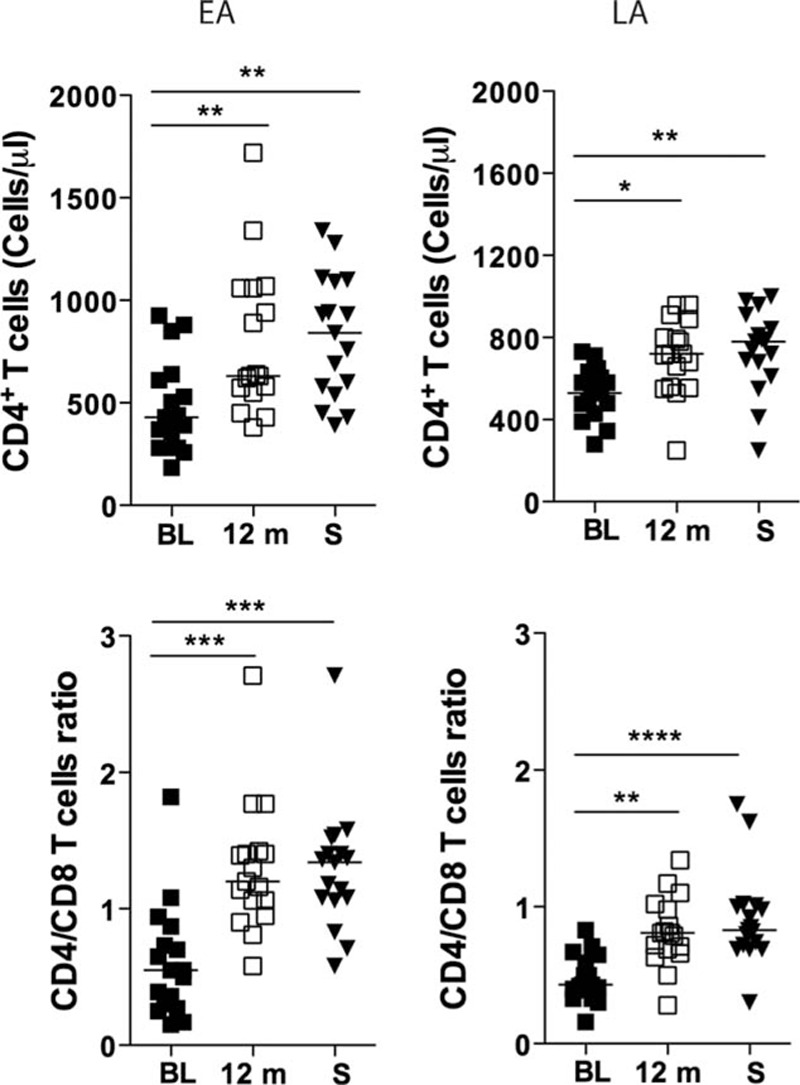
CD4+ T-cell counts and CD4+/CD8+ ratio in the blood of individuals treated during primary (EA) and chronic (LA) phases of HIV-1 infection. CD4+ T-cell counts (cells/μL) and the ratio between CD4+ T cells and CD8+ T cells (CD4+/CD8+ ratio) were measured in the blood of subjects belonging to both EA (n = 17) and LA (n = 17) study groups. The measurements were performed before ART initiation (baseline [BL]), at 12 months (12 m) after ART and at sampling (S). The statistical difference in the values between the time points was calculated using ANOVA. Median values are shown in the figures. ^∗^*P* < 0.05, ^∗∗^*P* < 0.01, ^∗∗∗^*P* < 0.001, and ^∗∗∗∗^*P* < 0.0001. ANOVA, analysis of variance; ART, antiretroviral therapy; EA, early ART; LA, late ART.

The absolute CD4+ T-cell number (cells/μL blood) and the CD4+/CD8+ T-cell ratio were assessed at initiation of therapy, at 12 months from ART initiation, and at time of sampling of the specimens for both the EA and LA groups. These data were obtained from available clinical records and presented in Fig. 1 as part of the results.

Blood (30–40 mL) was collected aseptically from patients and controls in ethylenediaminetetraacetic acid-containing tubes; PBMCs were isolated and stored in 90% fetal bovine serum (Sigma, St. Louis MO) and 10% dimethyl sulfoxide (Sigma) in liquid nitrogen (−160°C) until further analyses were conducted. The plasma specimens were frozen at −80°C until the analyses were conducted.

The ethical committee at Karolinska Institutet approved the study, and written informed consent was obtained from all individuals included.

### 2.2 Measurement of soluble inflammation parameters in serum

We measured the concentration of 9 inflammatory parameters in the plasma of HIV-1-infected patients (EA and LA) and controls. The inflammatory markers included interleukin (IL)-6, IL-8, tumor necrosis factor (TNF), C-reactive protein (CRP), IFN-gamma inducible protein 10 (IP-10), sCD14, β-2 microglobulin (β2M), CXCL1, and IL-21. In addition, we measured hyaluronic acid (HA) as a marker of fibrosis.

The soluble markers were assessed by commercially available ELISA kits. The β2M ELISA was from Thermo Scientific (Waltham, MS), the IL-21 ELISA from Mabtech (Nacka, Stockholm, Sweden), and the remaining ELISA assays were purchased from R&D (Minneapolis, MN). The plasma dilution to be used in the individual assays was evaluated before the assessment of the plasma obtained from the patients and controls. The ELISA assays were run according to the instructions from the manufacturer.

### 2.3 Measurement of total PBMC HIV-1 DNA

The PBMC DNA was obtained by manual extraction with the High Pure Viral Nucleic Acid Kit (Roche). Total PBMC HIV-1 DNA was quantified by using a homemade Taqman real-time assay targeting a highly conserved region of the long terminal repeat gene.^[24]^ To correct for minor deviations from the expected DNA input, total HIV-1 DNA copy numbers were normalized on a beta-globin standard curve and expressed as copies per million PBMCs. The beta-globin standard curve was made of 5 points (10, 100, 1000, 10,000, 100,000 copies) of human DNA derived from Sup-T1 cells. Primer P222 (5’-AGGGCCTCACCACCAACTT-3’) and P223 (5’-GCACCTGACTCCTGAGGAGAA-3’) were used together with probe P672 (5’-VIC ATCCACGTTCACCTTGCCCCACA-3’-TAMRA) to amplify the beta-globin control target. To ensure accurate normalization, HIV-1 and beta-globin DNA were amplified in the same reaction tube. The standard curve for quantification of total HIV-1 DNA was obtained from serial dilutions of the pNL4–3 plasmid containing the full HIV-1 genome. To mimic HIV-1 DNA quantification in clinical samples, the pNL4-3 plasmid was diluted using a solution containing 50 ng/μL of human DNA. The pNL4-3 standard curve was normalized on the calibration curve of the international quality control PCR Reference Kit/95 series (NIBSC). The reaction mixture included 750 ng of DNA extract as determined spectrophotometrically, 10 μL Premix Ex Taq Probe qPCR (Takara), 7.5 pmol of each primer, and 2.5 pmol of the probe in a final volume of 20 μL. Reactions were run in a Light Cycler 96 system (Roche) for 45 cycles each including 15 seconds at 95°C and 1 minute at 57°C.

The inter-run reproducibility of the assay was preliminary determined by running 9 independent runs with both standard curves. The inter-run coefficient of variation for HIV-1 DNA was 1.96%, 3.22%, 3.52%, and 2.94% with 10, 100, 1000, and 10,000 nominal target copies, respectively. The inter-run coefficient of variation for beta-globin was 9.20%, 3.02%, 4.30%, 5.37%, and 7.14% with 10, 100, 1000, 10,000, and 100,000 nominal target copies, respectively. The limit of detection was 10 HIV-1 DNA copies per million PBMCs. Samples with HIV-1 DNA values below 10 copies/million PBMCs were analyzed in triplicate to increase the accuracy of the result, with the final value reported as the mean of the 3 individual tests.

### 2.4 Immunostainings of T-cell subpopulations

Isolated PBMCs were stained using fluorochrome-conjugated antibodies in different combinations: anti-CD3 (UCHT1), anti-CD4 (Clone L200 or RPA-T4), anti-CD8 (SK1 or RPA-T8), anti-CD28 (CD28.2), anti-CD38 (HIT2), anti-CD45RA (HI100), anti-CD57 (NK1), anti-CD127 (HIL-7R-M21), anti-PD-1 (EH12.1 or MIH4), anti-CCR7 (3D12), and anti-HLA-DR (G46-6), all from BD Biosciences (CA). Ki67 (Clone MIB-1) was from Dako Denmark A/S (Glostrup, Denmark). LIVE/DEAD Fixable Near-IR kit (Life Technologies Europe BV, Stockholm, Sweden) was used to exclude dead cells from the analyses. Stained cells were washed 3 times with phosphate-buffered saline before being fixed in 2% paraformaldehyde. All antibodies were used at the concentrations determined after titration experiments. Matched isotype controls or fluorescence minus one (FMO) were used to set the gating strategies. Fluorescence intensities were measured with LSRII (BD), and data were analyzed using FlowJo, version 9.4.11 (Tree star, OR).

T-cell subsets were gated out using the following antibody combinations. CD4+ T-cell subsets: total CD4+ T cells (CD3+CD4+CD8−), CD4+ terminally differentiated effector memory (TEMRA) (CD3+CD4+CD8−CD45RA+CCR7−), CD4+ naive (CD3+CD4+CD8−CD45RA+CCR7+), CD4+ effector memory (EM) (CD3+CD4+CD8-CD45RA−CCR7−), and CD4+ central memory (CM) (CD3+CD4+CD8−CD45RA−CCR7+). CD8+ T-cell subsets: total CD8+ T cells (CD3+CD4−CD8+), CD8+ TEMRA cells (CD3+CD4−CD8+CD45RA+CCR7−), CD8+ naive (CD3+CD4−CD8+CD45RA+CCR7+), CD8+ EM (CD3+CD4-CD8+CD45RA−CCR7−), and CD8+ CM (CD3+CD4-CD8+CD45RA−CCR7+).

### 2.5 Statistical analyses

Data were tested for normal distribution by Kolmogov–Smirnov test, and analyses were performed using 1-way analysis of variance (ANOVA) with Dunnes posttest when different patient groups were compared. Correlation coefficients and their significance were calculated using Spearman rank correlation. Differences with *P* < 0.05 were considered significant. Prism version 5.0a (GraphPad Software, La Jolla, CA) software was used for the analyses.

## 3 Results

### 3.1 Description of the cohorts and clinical findings at baseline before ART, at 12 months from initiation of ART, and at the time of specimen collection

The number of CD4+ T cells in the blood of HIV-1-infected patients (EA and LA) at different time points was available from clinical records. Fig. 1 shows the absolute number (cells/μL blood) and the CD4+/CD8+ T-cell ratio for both the EA and LA groups at initiation of therapy, at 12 months from ART initiation, and at time of sampling of the specimens included in the present study. As shown in Fig. 1, the absolute number of CD4+ T cells increased in both the EA and LA groups during therapy. In the EA group, the absolute CD4+ T-cell counts increased from a median value of 430 cells/μL blood at initiation of therapy, to 630 at 12 months from ART initiation (*P* < 0.01), and 840 at sampling (*P* < 0.01). A similar picture was observed for the LA group with an increase of the CD4+ T-cell counts from 530 cells/μL blood at initiation of therapy to 720 at 12 months of ART (*P* < 0.05) and to 780 at sampling (*P* < 0.01).

The median CD4+/CD8+ cell ratio increased over time in both the EA and LA groups; however, the increase was more significant in the EA group where the ratio increased from 0.55 at therapy baseline to 1.2 at 12 months of ART (*P* < 0.001) and to 1.34 at sampling (*P* < 0.001). The corresponding values for the LA group were 0.43 (baseline), 0.81 (12 months; *P* < 0.01), and 0.83 (*P* < 0.0001); thus the median CD4+/CD8+ cell ratio never reached the value of 1 or above in the group that received ART during the chronic phase of HIV-1 infection.

### 3.2 Soluble markers of immune activation and inflammation in plasma

We measured the levels of soluble markers of inflammation in the plasma of EA, LA, and control groups (Supplementary Fig. 1). The plasmatic levels of the markers CRP, IL-6, IL-8, IL-21, TNF, IP-10, CXCL1, and HA were not statistically different between the 3 groups. The levels of sCD14, however, were significantly higher in the group of EA patients (median 1.46 μg/mL) as compared with the control group (0.82 μg/mL; *P* < 0.05). In addition, the levels of β2M were higher in the LA group (1.56 μg/mL) as compared with the control group (1.10 μg/mL; *P* < 0.01).

We correlated the levels of soluble markers of inflammation with the length of treatment and with the absolute number and frequency of CD4+ T cells, and the CD4+/CD8+ ratio at the time of sampling. The only parameters which showed a moderate level of correlation with the length of treatment were the levels of β2M in the EA group (*r* = 0.5, *P* < 0.05) and the levels of sCD14 in the LA group (*r* = −0.5, *P* < 0.05; results not shown).

### 3.3 Frequencies of total CD4+ T cells and CD8+ T cells and their subpopulations

**Figure 2:**
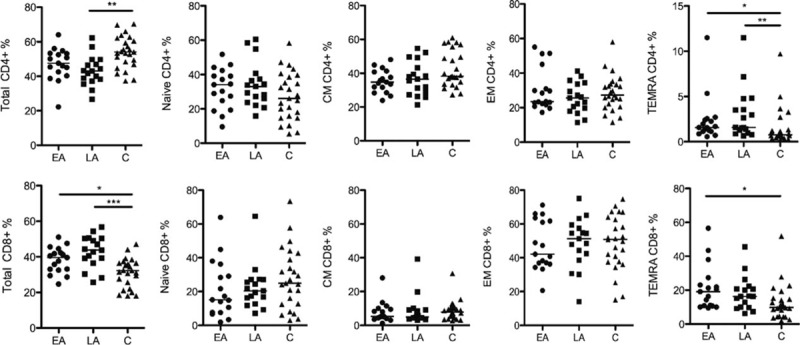
Frequency of CD4+ and CD8+ T cells and their subsets in PBMCs. The frequency of total CD4+ and CD8+ T cells, among CD3+ T cells, and the T-cell subsets naive, CM, EM, and TEMRA calculated out of total CD4+ or total CD8+ T cells are shown. The data are presented for all 3 groups studied: EA (n = 17), LA (n = 17), and C (n = 25). The line in the figures represents median values and the statistics were calculated using ANOVA. ^∗^*P* < 0.05, ^∗∗^*P* < 0.01, and ^∗∗∗^*P* < 0.001. ANOVA, analysis of variance; ART, antiretroviral therapy; CM, central memory; EA, early ART; EM, effector memory; LA, late ART; TEMRA, terminally differentiated effector memory cells.

We assessed the frequencies of CD4+ and CD8+ T cells and subpopulations of CD4+ and CD8+ T cells in blood specimens from EA, LA, and control subjects. The gating strategy for identification of the different T-cell subpopulations is presented in supplementary Fig. 2. The frequency of the total population of CD4+ T cells (Fig. 2) was lower in the LA group as compared with controls (median 43.1% vs 54.1%; *P* < 0.01); the frequency of CD4+ T cells in the EA group was also lower in comparison with the control group (median 47.6% vs 54.1%), but this difference was not statistically significant. The frequency of total CD8+ T cells, on the other hand, was higher in both groups of EA and LA as compared with controls (39.6% vs 32.2%, and 43.8% vs 32.2%; *P* < 0.05 and *P* < 0.001, respectively).

As illustrated in Fig. 2, the frequency of naïve, CM, and EM CD4+ and CD8+ T cells did not differ between the 3 groups studied. However, the median frequencies of terminally differentiated (TEMRA) CD4+ T cells were increased in both EA (1.57%) and LA (1.58%) groups as compared with healthy controls (0.74%; *P* < 0.05 and *P* < 0.01, respectively). The frequency of TEMRA CD8+ cells varied between the EA and control groups (median 19.1% vs 9.8%; *P* < 0.05). No differences were detected for the frequencies of the studied CD4+ and CD8+ T-cell populations between the EA and LA groups.

### 3.4 Expression of markers of activation, exhaustion, and differentiation on selected populations of T cells

The expression of different CD4+ and CD8+ T cells markers associated with HIV pathology were studied. These included activation (HLA-DR and CD38), terminal differentiation (CD127), and exhaustion (PD-1, CD28, CD57) markers (Tables 1 and 2).

**Table 1:**
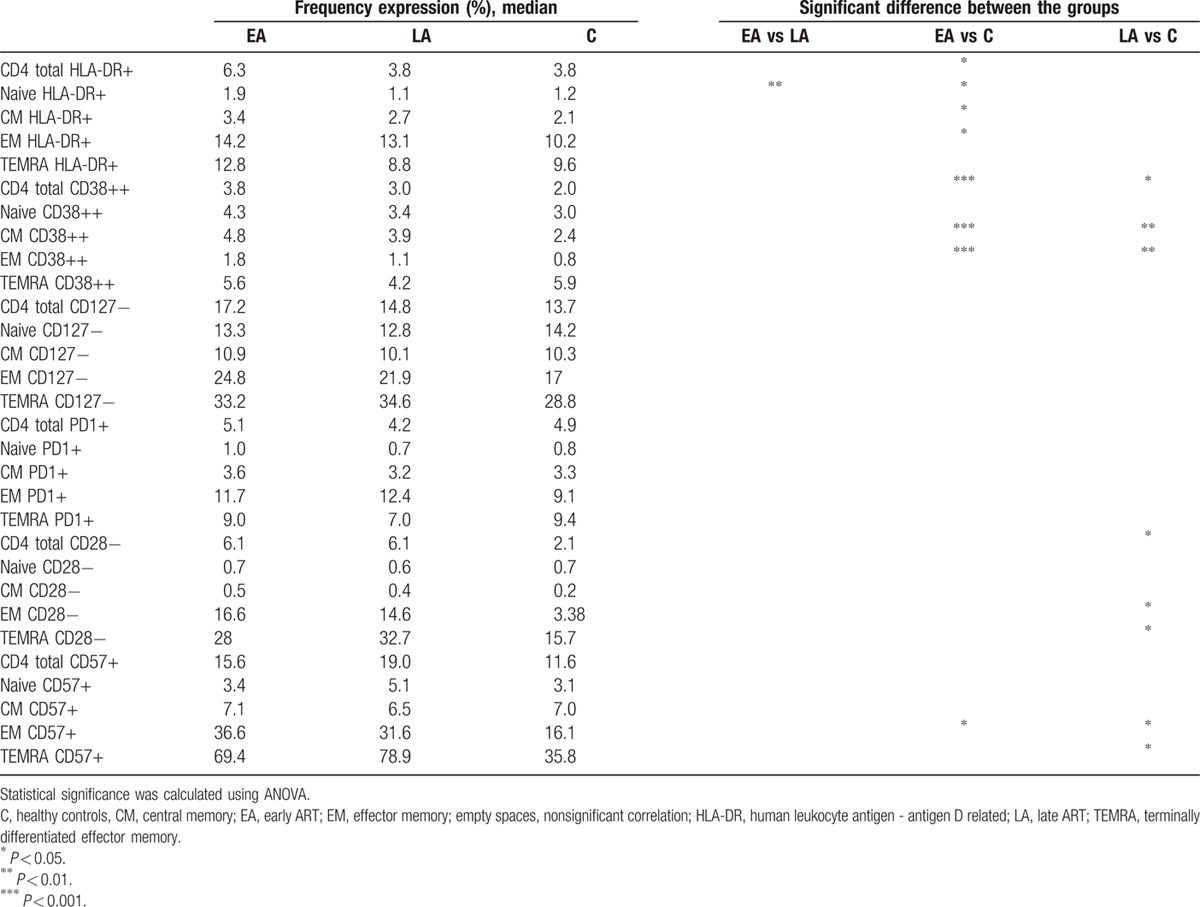
Expression of surface markers on CD4+ T cells and subpopulations.

**Table 2:**
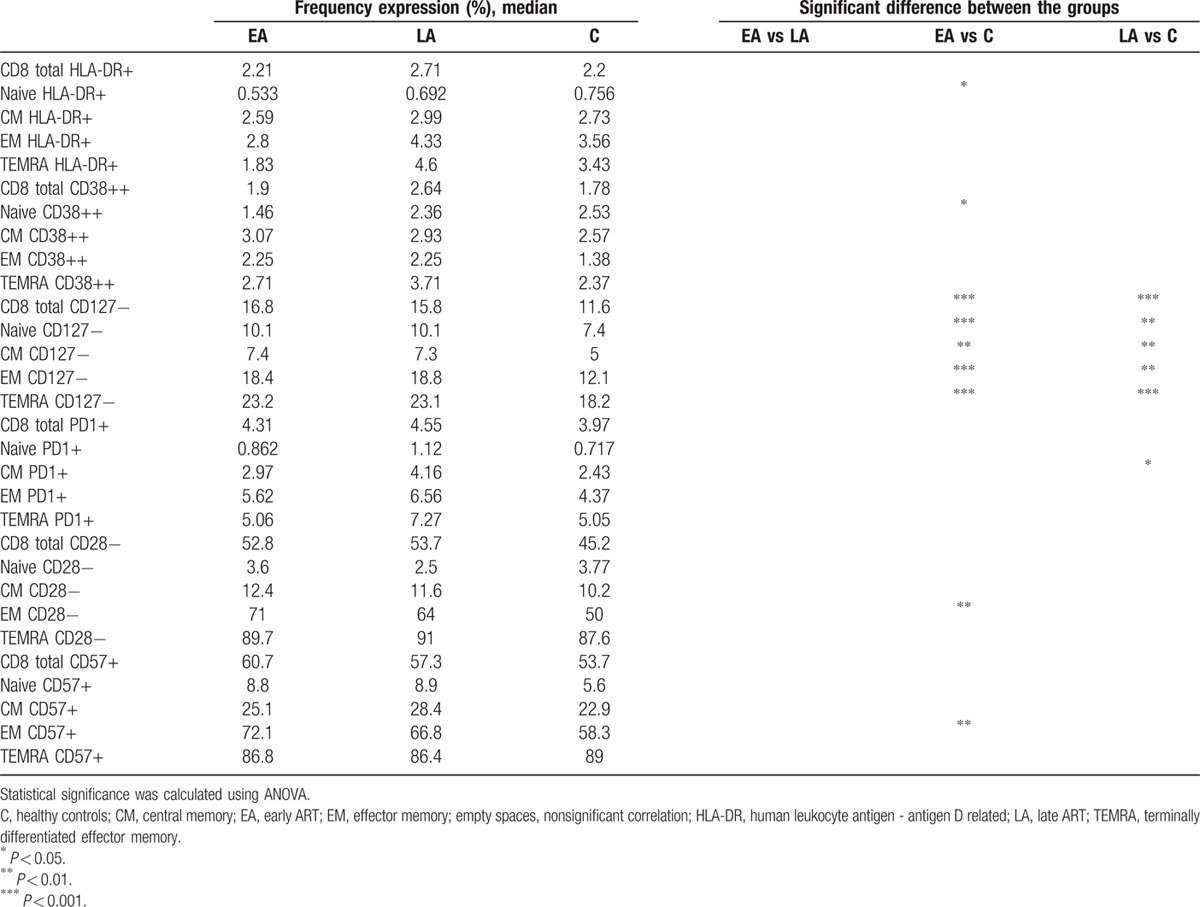
Expression of surface markers on CD8+ T cells and subpopulations.

A higher frequency of total CD4+ T cells, and also naïve, CM, and EM CD4+ T cells from the EA group (Table 1) were HLA-DR+ (*P* < 0.05) compared with the corresponding cell populations from controls. Among the total, CM, and EM CD4+ T cells from the EA a higher frequency of cells were CD38++ as compared with cells from controls (*P* < 0.001). As shown in Table 1, the frequency of EM CD4+ T cells expressing the exhaustion marker CD57 was higher in the EA group compared with the control group (*P* < 0.05).

Several differences were observed between the LA and control groups in relation to the frequencies of CD38++, CD28−, and CD57+ T cells (Table 1). The frequencies of total, CM, and EM CD38++ CD4+ T cells were higher in the LA group as compared with controls (total, *P* < 0.05; CM cells, *P* < 0.01; and EM cells, *P* < 0.01). A larger frequency of CD28− total, EM, and TEMRA CD4+ T cells were identified in the LA group as compared with the control group (*P* < 0.05 for all CD4+ T-cell groups). The frequency of CD57+ EM and TEMRA CD4+ T cells was higher in the LA versus control group (*P* < 0.05). When comparing the 2 groups of HIV-1-infected individuals, a higher frequency of HLA-DR+ naïve CD4+ T cells in circulation was found in the EA group as compared with the LA group (*P* < 0.01).

As for the surface markers on the CD8+ T-cell populations (Table 2), the lack of expression of the CD127 alpha chain of the IL-7R significantly differentiated cells from both the EA and LA HIV-1-infected population, in relation to the control group. A higher frequency of CD127− cells was found among the total CD8+ T cells and in all subpopulations of CD8+ T cells of EA and LA as compared with the control group (significance ranging between *P* < 0.01 and *P* < 0.001; Table 2).

A lower frequency of HLA-DR+ and CD38++ naïve CD8+ T cells was found in the EA group as compared with the control group (*P* < 0.05). Furthermore, a larger frequency of EM CD8+ T cells from the EA group was CD28− and CD57+ (*P* < 0.01) as compared with controls, and a larger frequency of CM CD8+ T cells from the LA group expressed the PD-1 marker (*P* < 0.05).

**Figure 3:**
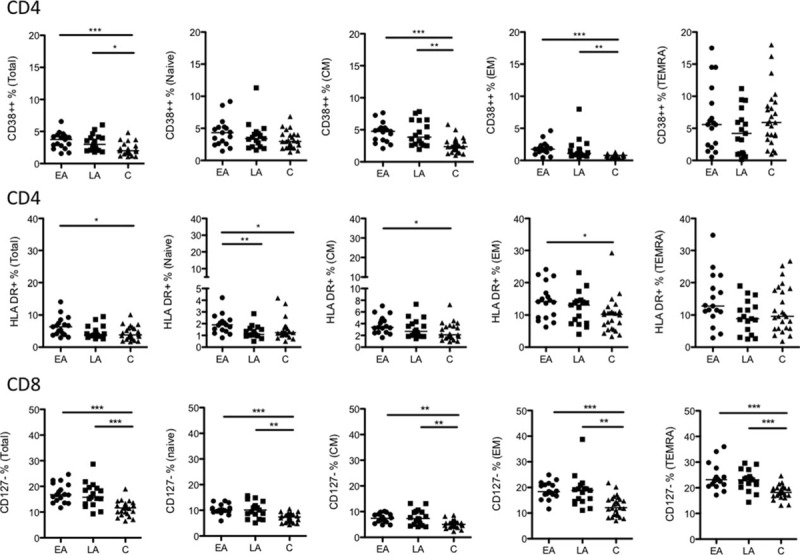
Frequency of CD38++ CD4+, HLA-DR+ CD4+, and CD127− CD8+ T-cell subsets. The frequency of CD38++ and HLA-DR+ cells among total, naive, CM, EM, and TEMRA CD4+ T cells is shown together with the frequency of CD127− total, naive, CM, EM, and TEMRA CD8+ T cells. The data are presented for the 3 groups studied: EA (n = 17), LA (n = 17), and C (n = 25). The line in the figures represents the median values and the statistics were calculated using ANOVA. ^∗^*P* < 0.05, ^∗∗^*P* < 0.01, and ^∗∗∗^*P* < 0.001. ANOVA, analysis of variance; ART, antiretroviral therapy; CM, central memory; EA, early ART; EM, effector memory; HLA-DR, human leukocyte antigen - antigen D related; LA, late ART; TEMRA, terminally differentiated effector memory cells.

To visualize the expression of individual markers which significantly distinguished CD4+ and CD8+ T-cell populations in HIV-1-infected patients and controls, flow cytometry results are shown (Fig. 3).

### 3.5 The frequency of CD4+ and CD8+ T-cell populations expressing different surface markers in relation to CD4+ T-cell counts, CD4+/CD8+ ratio, and treatment length

Comparison of the median expression for the different markers on CD4+ and CD8+ T cells revealed that the expression of 3 sets of markers regularly distinguish T-cell subpopulations: HLA-DR+ distinguished EA CD4+ T cells from LA, and also EA from controls; CD38++ distinguished CD4 T cells and CD127− distinguished CD8 T cells of both groups from controls. Further analyses of these 3 markers were conducted, in which their expression on the different subpopulations of EA and LA T cells were correlated with CD4+ T-cell counts, CD4+/CD8+ ratio, and treatment length at the time of sampling. These correlations are shown in Tables 3 and 4 for the specimens obtained from both EA and LA patients.

**Table 3:**
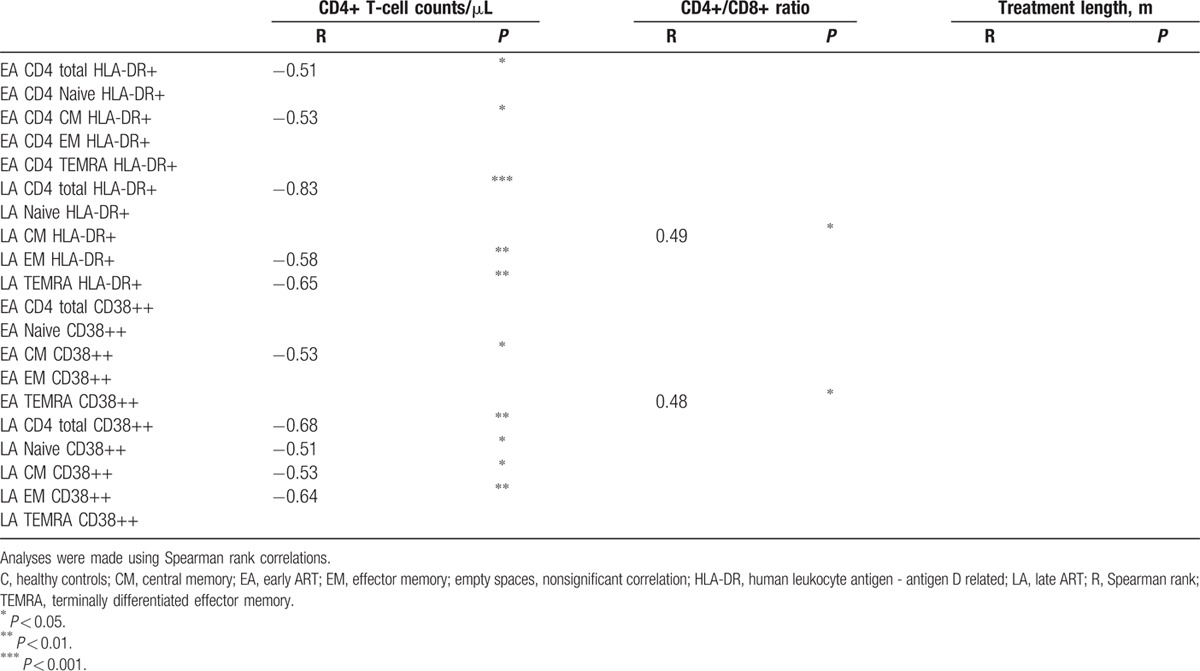
Correlations of CD4+ T-cell populations expressing different surface markers with CD4+ T-cell counts, CD4+/CD8+ ratio, and treatment length.

**Table 4:**
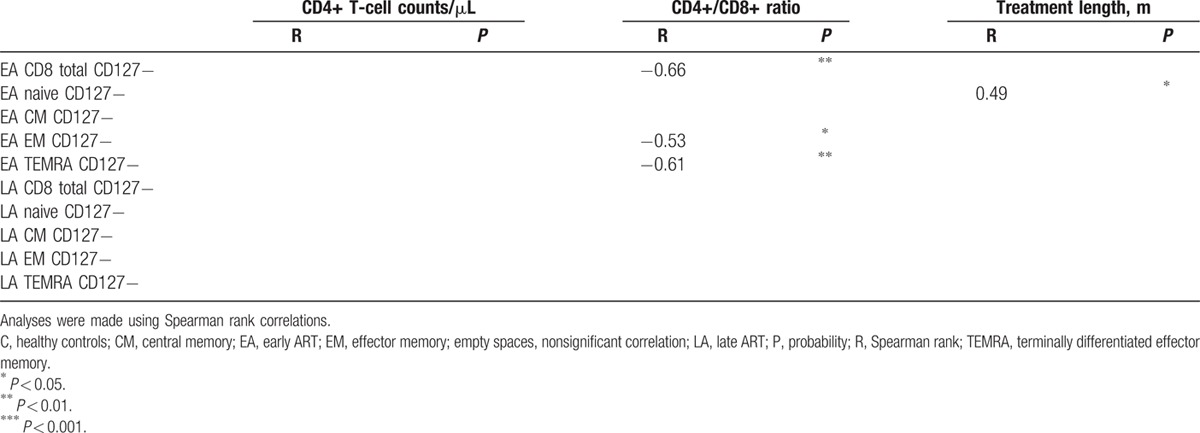
Correlations of CD8+ T-cell populations negative for CD127 expression with CD4+ T-cell counts, CD4+/CD8+ ratio, and treatment length.

Table 3 shows that in the EA and LA groups, the frequency of HLA-DR+ and CD38++ in CD4+ T-cell subpopulations negatively correlated with the number of CD4+ T cells/μL. This correlation was noted for most CD4+ T-cell subpopulations HLA-DR+ and CD38++ in the LA group (with p ranging between 0.05 and 0.0001), and also for the EA group HLA-DR+ total CD4+ T cells (*P* < 0.05), HLA-DR+ CM CD4+ T cells (*P* < 0.05), and CD38++ CM CD4+ T cells (*P* < 0.05). The graphs for the significant correlations presented in Table 3 are shown in Supplementary Fig. 3.

Table 4 shows the correlation of CD8+ T-cell subpopulations from EA and LA-negative for CD127 expression with CD4+ T-cell counts, CD4+/CD8+ ratio, and treatment length. The frequency of CD127− CD8+ T-cell subpopulations from the EA group inversely correlated with the CD4+/CD8+ ratio, whereas lack of CD127 expression on CD8+ T-cell subpopulations from the LA group did not correlate with this parameter. A correlation was found between the frequency of CD127− naïve CD8+ T cells of EA and treatment length (*P* < 0.05). The graphs for the significant correlations presented in Table 4 are shown in Supplementary Fig. 4.

### 3.6 Proliferation of T cells

**Figure 4:**
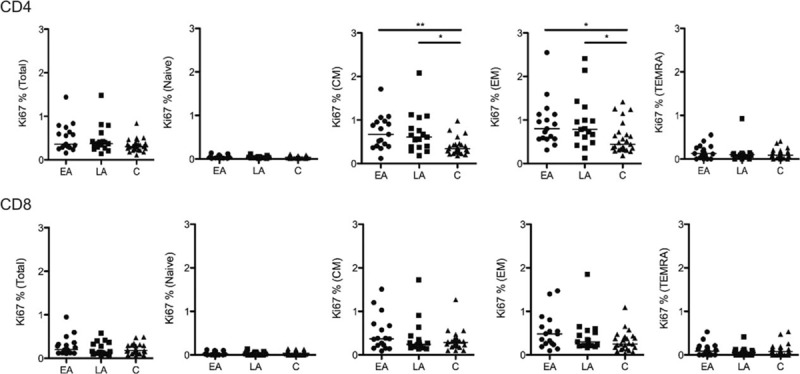
The frequency of Ki67 expressing CD4+ and CD8+ T cells. The frequency of Ki67-positive total CD4+ and CD8+ T cells and CD4+ T cells and CD8+ T-cell subsets (naive, CM, EM, and TEMRA) in the 3 groups studied are shown. The line represents median values and the differences between the groups have been calculated using ANOVA. ^∗^*P* < 0.05 and ^∗∗^*P* < 0.01. ANOVA, analysis of variance; ART, antiretroviral therapy; CM, central memory; EM, effector memory; TEMRA, terminally differentiated effector memory cells.

The expression of the intracellular Ki67 marker is widely used to characterize proliferating cells. Fig. 4 illustrates that a larger frequency of CM CD4+ T cells from EA and LA groups expressed Ki67 as compared with the control group (*P* < 0.01 and *P* < 0.05, respectively). The same observation was made for the EM CD4+ T cells of EA and LA among which a higher frequency expressed Ki67 than T cells from the controls (*P* < 0.05 for both).

We examined the correlation between the frequency of CD4+ and CD8+ T-cell subpopulations expressing Ki67+ and the CD4+ T-cell counts, the CD4+/CD8+ ratio, and treatment length. As shown in Table 5, an indirect correlation was found between the CD4+ T-cell counts and the frequency of Ki67+ total CD4+ T cells (*P* < 0.001), Ki67+ CM CD4+ T cells (*P* < 0.05), and Ki67+ EM CD4+ T cells (*P* < 0.05) from LA patients. Similarly, CD4+ T-cell counts from the same LA group inversely correlated with frequency of Ki67+ total CD8+ T cells (*P* < 0.05), Ki67+ CM CD8+ T cells (*P* < 0.01), and Ki67+ EM CD4+ T cells (*P* < 0.05). In addition, a direct correlation was found between the CD4+/CD8+ ratio and the frequency of Ki67+ TEMRA CD4+ T cells isolated from EA patients (*P* < 0.01).

**Table 5:**
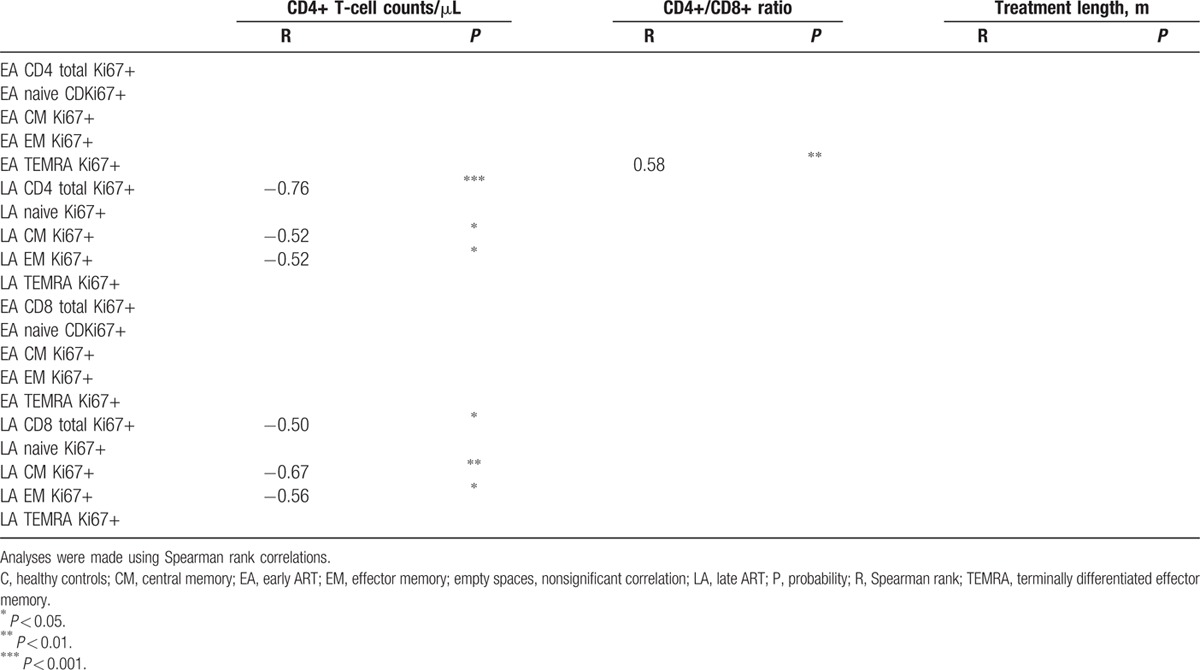
Correlations of CD4+ and CD8+ T-cell populations expressing Ki67 surface marker with CD4+ T-cell counts/μL, CD4+/CD8+ ratio, and treatment length.

### 3.7 Quantification of HIV-1 DNA in PBMCs and correlation with T-cell subpopulations

**Figure 5:**
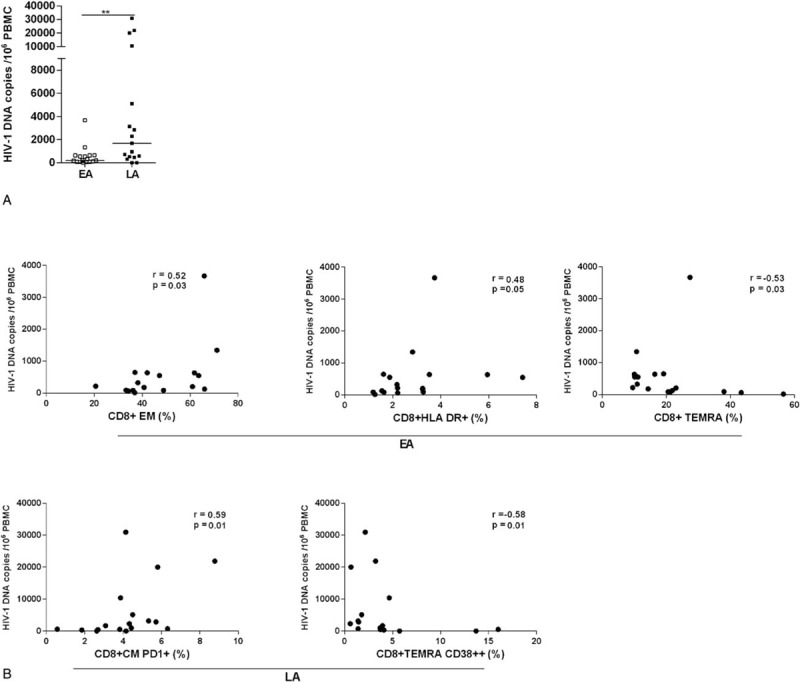
Size of total HIV-1 DNA copies and its correlation to T-cell subpopulations and surface markers. Copies of HIV-1 DNA in 10^6^ PBMCs from EA and LA patients (A). The line represents median values and the differences between the groups have been calculated using ANOVA (^∗∗^*P* < 0.01). In specimens from EA patients, the number of HIV-1 DNA copies was shown to directly correlate with the frequencies of CD8+ EM and CD8+ total HLA-DR+ T cells, whereas a negative correlation was shown between the virus reservoir and the frequency of CD8+ TEMRA T cells (B). In specimens from LA patients, the number of HIV-1 DNA copies, directly correlated to the frequency of CD8+ CM PD-1+ T cells and indirectly to the CD8+ TEMRA CD38++. Correlation coefficients and their significance were calculated using Spearman rank correlation. ANOVA, analysis of variance; ART, antiretroviral therapy; CM, central memory; EA, early ART; EM, effector memory; HLA-DR, human leukocyte antigen - antigen D related; LA, late ART; PD-1, programmed death 1; TEMRA, terminally differentiated effector memory cells.

The size of the virus reservoir was determined in EA and LA patients by quantification of total HIV-1 DNA in PBMCs. As previously reported by another group,^[3]^ the copies of HIV-1 DNA detected in PBMCs of patients treated during PHI are significantly lower than what is found in patients treated during the chronic phase of infection (median value for EA = 216 copies in 10^6^ PBMCs and for LA = 1681; *P* = 0.009) (Fig. 5A). HIV-1 DNA could not be detected in PBMCs from 3 patients, 1 in the EA group and 2 in the LA group.

We then proceeded to analyze whether the copies of total HIV-1 DNA found in the PBMCs of EA and LA patients correlated with the frequency of CD4+ and CD8+ T-cell subpopulations, also in relation to the different surface markers and Ki67 expression. The results of these correlations are illustrated in Fig. 5B. A positive correlation was found between the total HIV-1 DNA copies found in PBMCs of EA patients and the frequencies of CD8+ EM (*P* = 0.03) and total CD8+ HLA-DR+ T cells (*P* = 0.05), whereas an indirect correlation was detected with CD8+ TEMRA T cells (*P* = 0.03).

The copies of total HIV-1 DNA in the LA group correlated with the frequencies of total CD8+ CM PD-1+ (*P* = 0.01) and inversely with CD8+ TEMRA CD38++ (*P* = 0.01) T cells.

We could not find significant correlations between the total HIV-1 DNA and the frequencies of subpopulations of CD4+ T cells.

## 4 Discussion and conclusions

The principal aim of the study was to analyze whether the time point of ART initiation affects pathological expression of T-cell phenotypical markers reported to occur during HIV-1 infection and if ART initiation during the early phase of infection prevented phenotypical changes of T cells. Surprisingly, T-cell phenotypical changes detectable in patients who started ART very early are comparable with the dysfunctional phenotype identified in the group of HIV-1-infected individuals who started treatment during the chronic phase of infection. The major phenotypical changes identified were related to increased immune activation (HLA-DR+ and CD38++ mostly on CD4+ T cells), senescence (CD28− and CD57+ mostly on highly differentiated CD4+ and CD8+ T cells), and down-regulation of the alpha-chain of the IL-7 receptor CD127 (CD127− on CD8+ T cells and its subpopulations). Although conducted on a limited number of specimens, our study provides relevant information on phenotypical changes in T cells of patients treated early during HIV-1 infection. Opposite to the comparable dysfunctional T-cell phenotypes identified in EA and LA patients, the size of total HIV-1 DNA copies in PBMCs was significantly lower in EA patients.

The decline of CD4+ T cells during HIV-1 infection has been associated and correlated with the presence of activated T cells, cells characterized by the high expression of surface activation markers such as CD38 and HLA-DR; also in our study the expression of activation markers was inversely correlated to the CD4+ T-cell counts. Abnormally high activation levels of both CD4+ T cells and CD8+ T cells are independent predictors of CD4+ T-cell decline and progression to AIDS,^[25]^ and a continuous driving force fueling HIV-1 pathogenesis. In a study including a prospective cohort of HIV-1-infected individuals followed from acute infection, the rate of CD4+ T-cell loss was associated with the level of CD8+ T-cell activation.^[25]^ Decline of HIV-1 RNA in plasma upon ART is accompanied by a robust reduction, but not normalization, of immune activation.^[26,27]^ It is worrisome that high levels of immune activation, as measured by HLA-DR and CD38 expression, were found in our cohort of very early treated patients, especially in CD4+ T cells as compared with controls. It is of interest that the high frequency of CD38++ CD4+ T cells in the LA group inversely correlated to the CD4+ T-cell counts, whereas a lower degree of correlation between these parameters was found in the EA group. This result illustrates the relevance of further studying the CD38 marker as a driving force for HIV-1 immunopathogenesis.

In a recent study addressing biological markers predictive for HIV-1 rebound after treatment interruption in patients treated with ART from acute infection, Hurst et al^[28]^ found that expression of CD38 and HLA-DR on both CD4+ and CD8+ T cells correlated with the size of total HIV-1 DNA at PHI in a simple linear model. Furthermore, the expression of CD38 on CD8+ cells retained its predictive value in a multiple regression model.

We measured the size of total HIV-1 DNA in our cohorts of EA and LA patients. In line with what was previously reported,^[3]^ the number of HIV-1 DNA copies found in blood of EA patients was significantly lower than what was measured in LA patients, thus reiterating the relevance of starting ART as soon as possible, to reduce the number of virus reservoirs established during the early phase of HIV-1 infection. At the time of sampling, we found a direct correlation between HIV-1 DNA copies and HLA-DR+ CD8+ T cells in the EA group, whereas a direct correlation between the HIV-1 DNA copies and the frequency of PD-1+ CM CD8+ T cells was found in the LA group. It is possible to envisage that markers of exhaustion, PD-1, and increased immune activation, HLA-DR, directly correlate with the size of HIV-1 DNA as they may have a role in fueling virus replication by declining the quality of immune functions. We also found that the size of total HIV-1 DNA in blood indirectly correlated with CD8+ TEMRA in the EA group and with CD8+ TEMRA CD38++ T cells in the LA group. Further studies should be performed in this respect to clarify the role exerted by CD8+ TEMRA cells in controlling virus replication and establishment of virus reservoirs.

Taken together, the findings presented herein show that features of immune activation, exhaustion, and terminal differentiation are still present on CD4+ and CD8+ T cells in patients treated early after infection, and that their abnormal phenotypical features do not distinguish them from patients who started ART with a comparable number of CD4+ T cells during the chronic phase of infection. It is puzzling that these abnormalities are similar in the 2 groups despite a significant difference in the number of total HIV-1 DNA copies in PBMCs, which was lower in EA patients; however, it cannot be excluded that the size of virus reservoir may be different in relevant lymphoid tissues where the process of abnormal immune activation and terminal differentiation may take place despite successful ART.

Our study also shows that increased levels of proliferation, as measured by Ki67, are present at comparable levels in memory CD4+ T cells, both CM and EM, of EA and LA patients. We also found an indirect correlation between the number of CM and EM CD4+ and CD8+ T-cell subpopulations, and the frequency of Ki67-positive cells in the LA patients, reinforcing the knowledge that proliferation of T cells has a negative impact on the absolute CD4+ T-cell number. This result is important as it has recently been described that cell proliferation maintains a persistent HIV-1 DNA pool during effective ART.^[29]^ A negative correlation was found between the frequency of Ki67high Bcl2low CD8+ T cells and plasma viral load set point in a study aimed at elucidating how initial immune responses during early HIV-1 infection modulate the viral set point.^[30]^ In the specimens analyzed in our study, however, we could not find a correlation between the expression levels of Ki67 and the number of total HIV-1 DNA copies in PBMCs; differences in the design of the study and the cohorts of patients analyzed may account for these differences. One limitation of our study is that specimens to measure the size of total HIV-1 DNA copies in PBMCs were not available from the time when the patients initiated ART.

By measuring plasmatic soluble markers, significant levels of inflammation distinguishing the groups of HIV-1-infected individuals from one another could not be detected, likely due to the positive effect of ART in both groups of patients. However, the levels of sCD14—a molecule released from activated macrophages—were significantly higher in the group of EA patients as compared with the control group. Furthermore, the levels of β2M were higher in the LA group as compared with the control group, which in turn reflects activation of T and B cells.

Expression of CD127 has previously been reported to be lost on a large proportion of peripheral T cells in HIV-1-infected individuals, rendering these cells most probably unable to benefit from IL-7.^[12,31,32]^ Previous studies were performed on specimens obtained from cohorts of patients who received ART when CD4+ T-cell counts were lower than 350 cells/μL, thus likely experiencing a much higher degree of immunosuppression than the patients analyzed in the present study. The present results show that a significant proportion of the different CD8+ T-cell subpopulations from EA and LA lacked CD127 expression as compared with the control group. As IL-7 is a potent survival factor for T cells acting through the maintenance of basic cellular homeostasis and the regulation of antiapoptotic and proapoptotic Bcl-2 family member proteins,^[33]^ it can be envisaged that lack of CD127 expression may lead to impaired survival of CD8+ T cells despite ART initiation during the very early phase of infection. CD127-negative T cells in HIV-1-infected patients and older individuals have previously been shown to represent activated antigen-specific T-cell clones in late differentiation stages. This in turn implies that chronic antigenic stimulation may provide a driving force for CD127 down-regulation.^[31,34]^ However, it is unclear whether antigenic stimulation is the only mechanism leading to CD127 down-regulation, or if additional direct/indirect pathogenic mechanisms induced during HIV-1 infection are responsible for CD127 down-regulation.^[35]^ The mechanism for CD127 down-regulation during HIV-1 infection despite early and continuous treatment remains unknown. Therefore, studies aimed at characterizing the mechanism leading to this pathological finding should be pursued.

Fast and potent induction of CD8+ T-cell responses during PHI has been shown to impact the rate of early virus replication, because these cells seem to possess direct antiviral functions.^[30]^ Lack of CD127 expression on CD8+ T cells from primary infected patients, in combination with increased apoptosis, was a measure of impaired capacity of CD8+ T cells to fully suppress virus replication.^[30]^ Lécuroux et al^[36]^ previously studied the impact of ART treatment during PHI on CD127 expression on HIV-specific CD8+ T cells; their findings showed that CD127 expression on HIV-specific CD8+ T cells was very low during PHI, but increased significantly after ART initiation, reaching expression values and proliferative capacities comparable with what is found in HIV-infected nonprogressors. As CD127 and additional markers were not measured at the time of ART initiation during PHI in the EA group followed in our study, we cannot exclude the possibility that a large size of CD8+ T cells had lost CD127 expression at the time of seroconversion and that CD127 expression was increased at the time point examined in our study. As an alternative, it is possible that CD8+ T cells, even in presence of controlled virus replication, may acquire over time a CD127 negative phenotype, which may compromise sustainable virus suppression in patients interrupting ART. In this respect, it is interesting that the frequency of CD127– cells increased in all CD8+ T-cell populations, including naïve CD8+ T cells.

HIV-1 replication during very early phase of infection and persistence of the virus in reservoirs is likely the fueling motor that maintains an increased level of activation, exhaustion, and terminal differentiation of T cells. To identify a relevant combination of biomarkers which may be useful to predict patients who can become sustained posttreatment controllers is an important task currently undertaken by the HIV-1 scientific community.^[28]^ Markers of immune activation which were shown to correlate with the size of total HIV-1 DNA at primary HIV infection,^[28]^ did not predict the patients who would virus rebound at ART interruption, whereas levels of T-cell exhaustion before ART initiation were strongly associated with time to HIV-1 rebound. The results present herein suggest that CD127 should be further investigated as a biomarker to predict patients who can be successfully taken off therapy. In the present study, we found that the CD4+/CD8+ ratio increased significantly during ART in the group of EA patients, whereas the increase of CD4+/CD8+ ratio was lower in the LA group. The CD4+/CD8+ ratio is a clinical parameter used to assess the risk of possible complications including cardiovascular events or cancer. As the CD4+/CD8+ ratio was recently described to be a useful biomarker for assessment of combined CD4+ T-cell dysfunction in chronic HIV-1 disease, the relevance of this parameter to pin-point patients who may be taken off therapy should be further investigated.^[37]^

The patients included in this study receive ART under optimal clinical conditions, and Sweden is among the few countries worldwide where the 90-90-90 goals have been reached, where 90% of all people living with HIV will know their HIV status, 90% of all people with diagnosed HIV infection will receive sustained ART, and 90% of all people receiving ART will have viral suppression. The current Swedish policy is to treat HIV-1-infected patients as soon as they are referred to HIV-1 clinical units, independently of their CD4+ T-cell counts, and possibly already at the time of seroconversion. Increased knowledge in the field of ART treatment during early infection indicates that it might be beneficial to initiate treatment during this early phase of HIV-1 infection. The results present in this study confirm that early ART may confine the size of the virus reservoir and limit the inflammation process connected to high virus replication in the gut and other tissues.

In conclusion, although a lower size of virus reservoirs was found in EA patients, the results obtained from the phenotypical analyses of T-cell populations suggest precaution with interrupting ART in patients started on treatment during early infection. This is because some of the markers negatively linked to immune functions, including CD38, CD127, and T-cell proliferation, are comparable in the EA and LA-treated patients.

## Supplementary Material

Supplemental Digital Content
