# Supplementary material for: Dysfunctional phenotypes of CD4+ and CD8+ T cells are comparable in patients initiating ART during early or chronic HIV-1 infection
Source: Medicine (Baltimore). 2016 Jun 10;95(23):e3738. doi: 10.1097/MD.0000000000003738 (PMC4907649; doi:10.1097/MD.0000000000003738)

**Supplementary Figure 1. Quantification of soluble inflammation markers in plasma.**

The markers of inflammation CRP, sCD14, β2M, IL-6, IL-8, IL-21, TNF, IP-10, CXCL1 and the HA marker of fibrosis were measured in the plasma samples using ELISA. The data is presented for all three groups studied, EA (n=17), LA (n=17) and C (n=25). The difference between the groups was calculated using ANOVA and significant differences are shown in the figure. The lines in the figures represent median values. *p<0.05 and **p<0.01.


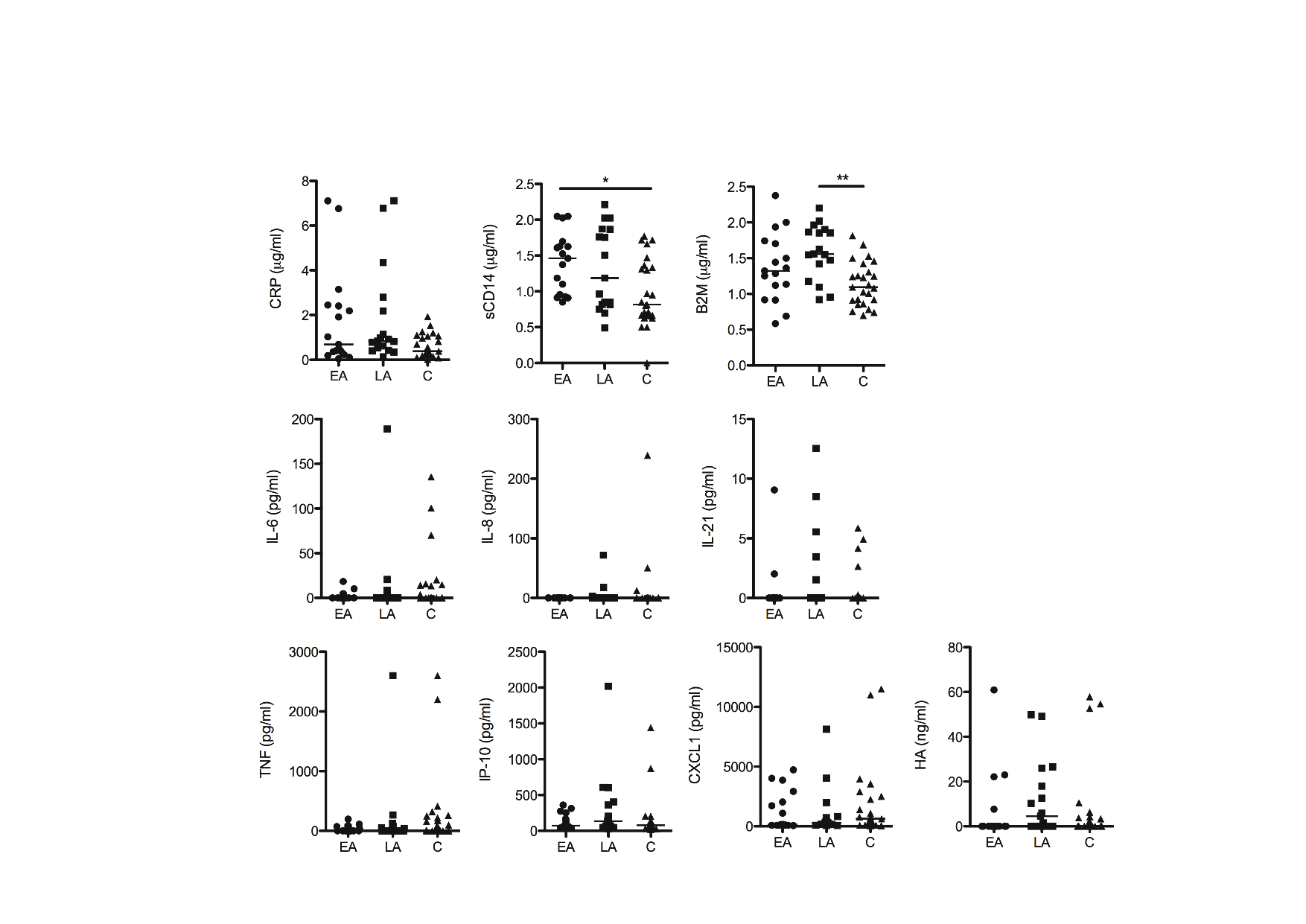


**Supplementary Figure 2. Gating strategy for CD4+ and CD8+ T cells and subpopulations.**

Total T cells were identified by CD3 surface expression after exclusion of dead cells and doublets. Gated CD3+ T cells were then divided into CD4+ and CD8+ T cell subsets. The sub-division of CD4+ and CD8+ T cells into TEMRA, Naïve, EM and CM sub-populations was achieved with CD45RA and CCR7 surface staining.

Characterization of CD28 positive or negative cells on the different CD4+ and CD8+ T cell sub-populations (naïve, CM, EM, TEMRA) is shown. The gating strategy for the CD28 is based on the fluorescence minus one (FMO) control.

**
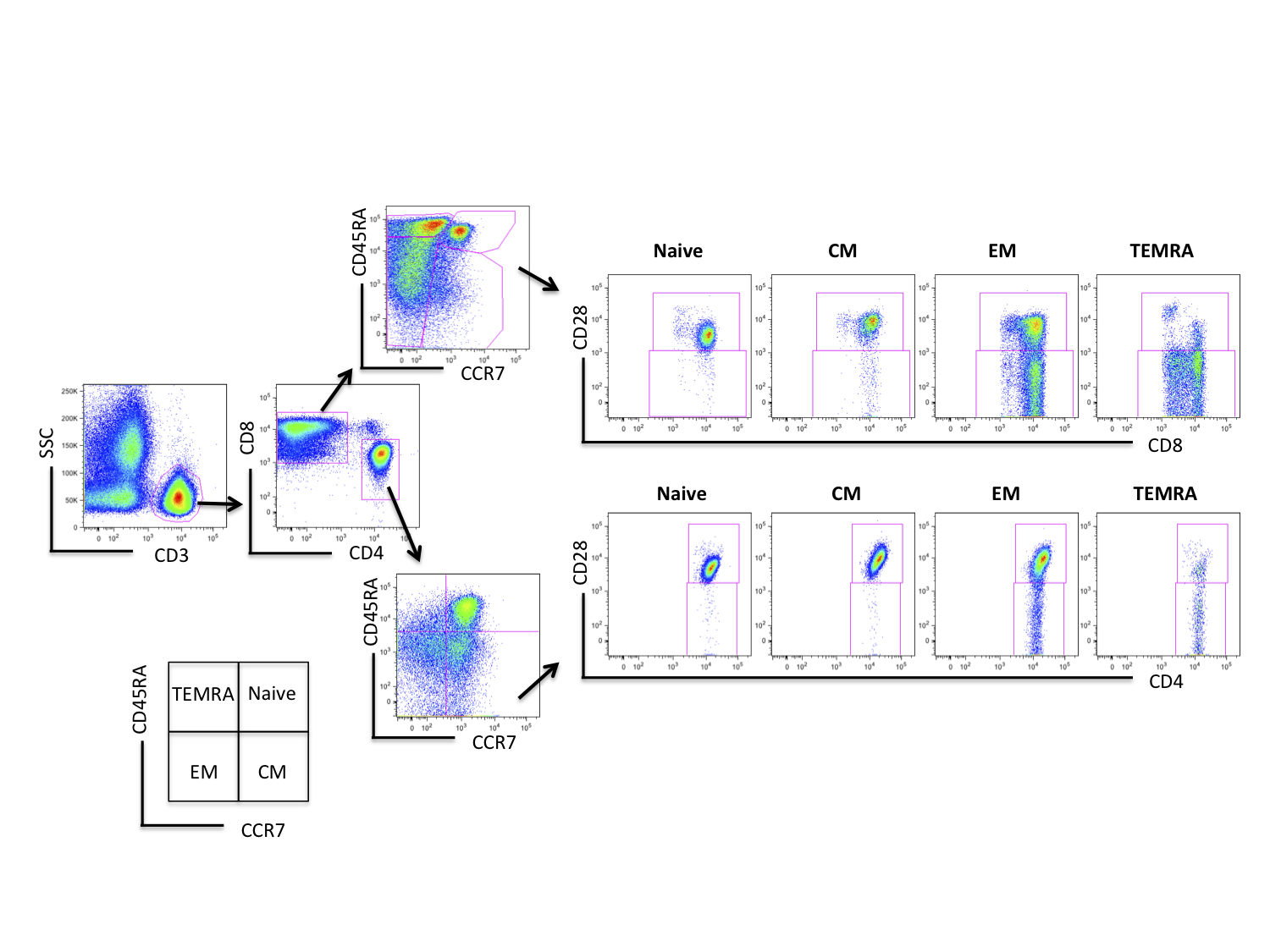
**

**Supplementary Figure 3. Graphs of significant correlations between CD4+ T cell populations expressing different surface markers with CD4 counts/μl and CD4/CD8 ratio.**

All correlations are also presented in table 3.

**
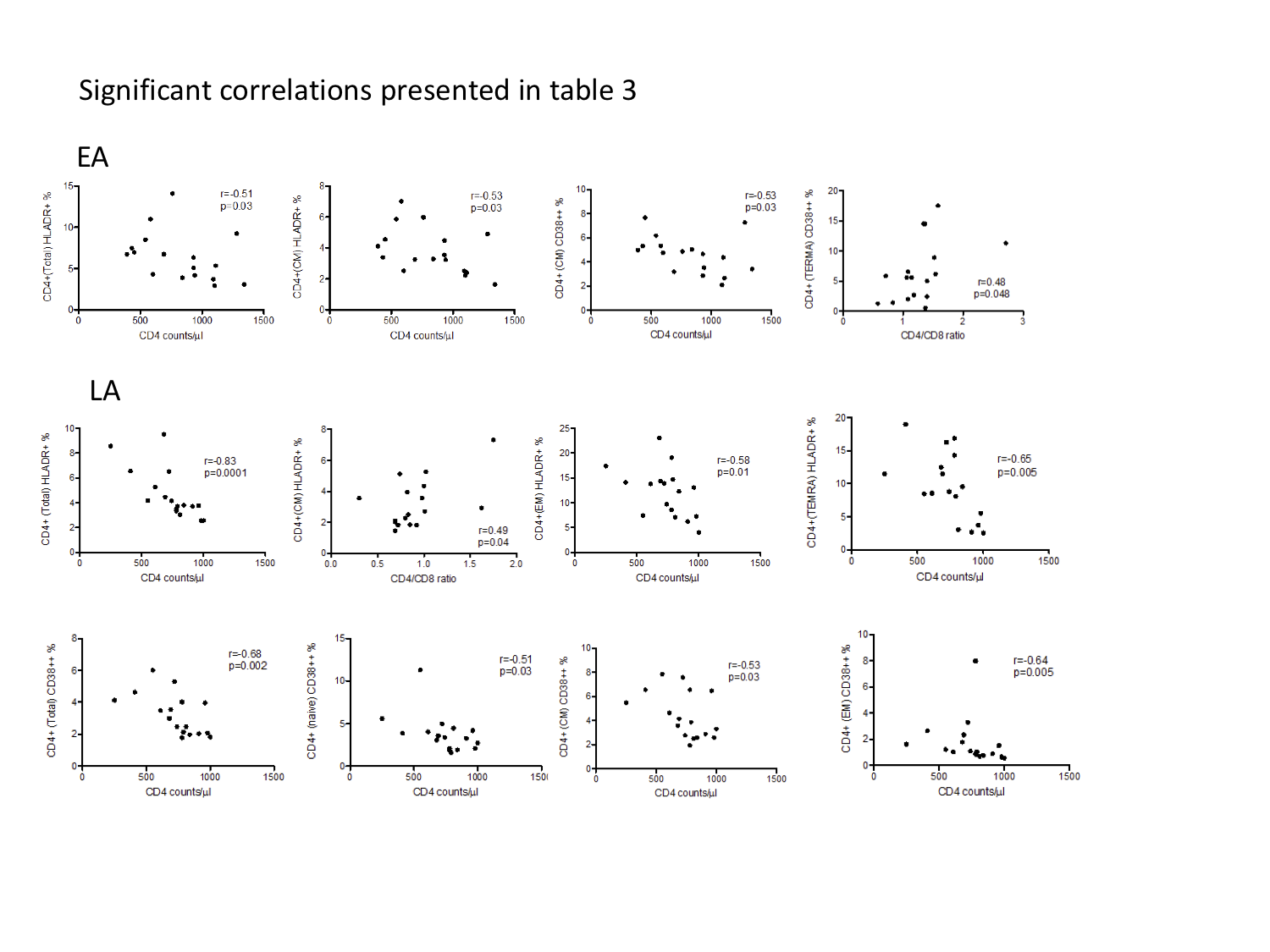
**

**Supplementary Figure 4. Graphs of significant correlations between CD8+ T cell populations expressing different surface markers with CD4 counts/μl and CD4/CD8 ratio.**

All correlations are also presented in table 4.


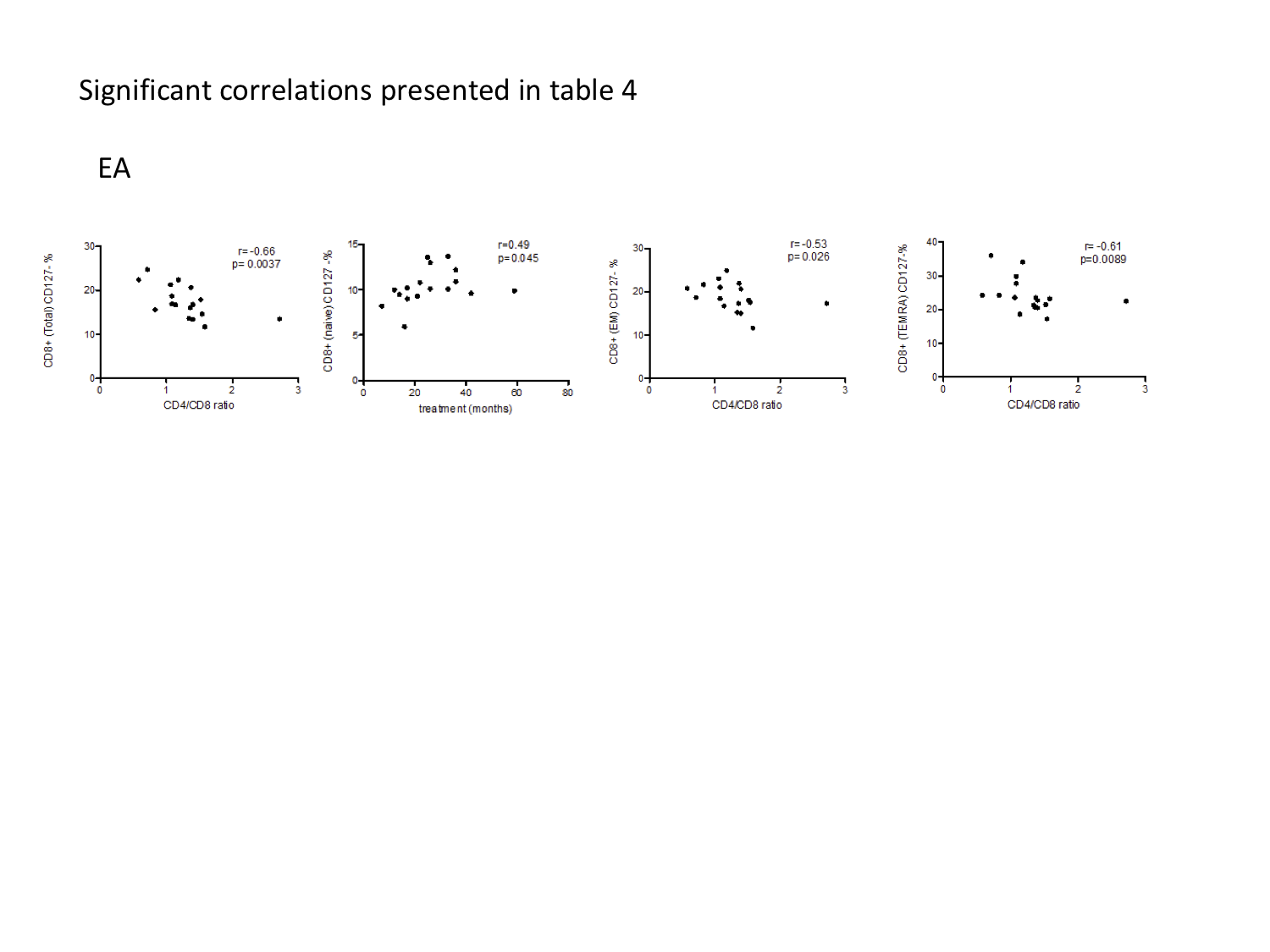

Supplement: Supplemental Digital Content [file medi-95-e3738-s001.doc]
